# Historical Manuscripts Analysis: A Deep Learning System for Writer Identification Using Intelligent Feature Selection with Vision Transformers

**DOI:** 10.3390/jimaging11060204

**Published:** 2025-06-19

**Authors:** Merouane Boudraa, Akram Bennour, Mouaaz Nahas, Rashiq Rafiq Marie, Mohammed Al-Sarem

**Affiliations:** 1 Laboratory of Mathematics, Informatics and Systems (LAMIS), Echahid Cheikh Larbi Tebessi University, Tebessa 12000, Algeria; merouane.boudraa@univ-tebessa.dz; 2 Department of Electrical Engineering, Umm Al-Qura University, Makkah 21955, Saudi Arabia; mmnahas@uqu.edu.sa; 3 College of Computer Science and Engineering, Taibah University, Medina 41477, Saudi Arabia; rmarie@taibahu.edu.sa; 4 Department of Information Technology, Aylol University College, Yarim 547, Yemen

**Keywords:** writer identification, historical manuscripts, intelligent features, deep learning, vision transformers, transfer learning

## Abstract

Identifying the scriptwriter in historical manuscripts is crucial for historians, providing valuable insights into historical contexts and aiding in solving historical mysteries. This research presents a robust deep learning system designed for classifying historical manuscripts by writer, employing intelligent feature selection and vision transformers. Our methodology meticulously investigates the efficacy of both handcrafted techniques for feature identification and deep learning architectures for classification tasks in writer identification. The initial preprocessing phase involves thorough document refinement using bilateral filtering for denoising and Otsu thresholding for binarization, ensuring document clarity and consistency for subsequent feature detection. We utilize the FAST detector for feature detection, extracting keypoints representing handwriting styles, followed by clustering with the k-means algorithm to obtain meaningful patches of uniform size. This strategic clustering minimizes redundancy and creates a comprehensive dataset ideal for deep learning classification tasks. Leveraging vision transformer models, our methodology effectively learns complex patterns and features from extracted patches, enabling precise identification of writers across historical manuscripts. This study pioneers the application of vision transformers in historical document analysis, showcasing superior performance on the “ICDAR 2017” dataset compared to state-of-the-art methods and affirming our approach as a robust tool for historical manuscript analysis.

## 1. Introduction

Understanding the identity of a writer within historical manuscripts is of profound significance for historians, serving as a key to unlocking the mysteries of the past. Revealing the authorship of manuscripts provides historians with invaluable insights into the historical contexts of their creation, shedding light on the social, cultural, and political landscapes of the era. This knowledge not only attributes specific texts to their authors but also enriches our understanding of the broader historical narrative, allowing historians to piece together a more accurate and nuanced portrayal of historical events. By identifying writers within historical manuscripts, historians gain a deeper understanding of the perspectives, beliefs, and ideologies prevalent during different historical periods. Moreover, writer identification facilitates the exploration of connections between authors, texts, and historical events, providing insights into the networks of intellectual and cultural exchange that shaped the course of history. Ultimately, delving into the identities of writers enriches our understanding of the past, revealing the diverse voices that have contributed to the tapestry of history and deepening our appreciation of the complexities of human experience across time and space.

Paleographers and historians have long grappled with the daunting task of determining the identities of writers within historical manuscripts, employing traditional manual methods involving visual examination of handwriting styles, meticulous comparison of letterforms, and detailed analysis of writing traits. These time-honored techniques, deeply rooted in centuries-old practices of textual scrutiny, have been fundamental to attributing manuscripts and establishing authorship. However, despite their historical precedence, manual approaches face inherent challenges that hinder their efficacy in the contemporary age. Firstly, they are exceedingly laborious and time-intensive, demanding meticulous scrutiny of each aspect of a manuscript’s script, which significantly prolongs the process of writer identification. Additionally, the subjective nature of manual analysis introduces variability and uncertainty, as human judgment and interpretation influence assessments of handwriting styles, potentially leading to discrepancies among analysts. Moreover, reliance on manual techniques increases the risk of errors, as human fallibility and the complexities of historical manuscripts may contribute to misinterpretations and inaccuracies in writer identification, even among experienced experts.

The transition from manual to automated methods for writer identification began in the late 20th century, coinciding with the advent of machine learning techniques [1,2,3,4,5]. This shift marked a significant departure from traditional approaches and ushered in a new era of computational analysis in historical manuscript research. Early automated efforts relied on basic statistical and pattern recognition algorithms to analyze handwriting features and classify writers based on distinct traits [6,7,8,9,10]. However, despite the potential of automation, these initial methods faced significant challenges. Variability in handwriting styles presented a major hurdle, with different writers exhibiting unique idiosyncrasies that made it challenging for algorithms to identify consistent patterns across manuscripts. Additionally, issues such as document degradation and noise further complicated the task, as poorly preserved manuscripts could obscure vital handwriting features, hindering accurate analysis. Moreover, the simplistic nature of early algorithms limited their ability to capture the full complexity of handwriting styles, leading to subpar performance and reduced accuracy in writer identification tasks. Despite these challenges, the advent of automated methods represented a significant step forward in the quest for more efficient and objective approaches to writer identification. While early endeavors may have faltered in overcoming the complexities of historical manuscript analysis, they laid the groundwork for subsequent advancements in machine learning and computational techniques, paving the way for more sophisticated and robust methodologies to emerge in the years to come.

The advent of deep learning methods has revolutionized writer identification, offering unparalleled performance and scalability compared to traditional machine learning approaches. Convolutional Neural Networks (CNNs) [11] and their variants have demonstrated remarkable success in extracting intricate patterns and features from handwritten texts [12,13,14,15]. One of the key advantages of deep learning lies in its ability to automatically learn hierarchical representations of data, enabling it to capture complex relationships and nuances within handwriting styles more effectively than conventional algorithms. Leveraging large datasets and sophisticated architectures, deep learning models can discern subtle variations in handwriting, even amidst noise and degradation in historical manuscripts [16,17,18,19,20]. However, challenges such as data scarcity, risk of overfitting, and computational complexity persist. Data scarcity, particularly in historical manuscript datasets, limits model training and generalization. The risk of overfitting necessitates careful regularization and validation strategies, while computational complexity demands substantial resources and time-intensive training procedures. Despite these challenges, the advent of deep learning has propelled writer identification research to unprecedented heights, promising further advancements and innovations in historical manuscript analysis and writer identification methodologies.

Recent advancements in computer vision have introduced vision transformers as a powerful alternative to traditional convolutional neural networks (CNNs) for image processing tasks. Vision transformers [21] employ a transformer architecture, utilizing self-attention mechanisms to capture long-range dependencies and contextual information within images more effectively than CNNs [22,23,24,25,26]. Inspired by these developments, our research aims to explore the potential of vision transformers and their variants such as Swin [27] and Deit [28] in historical manuscript writer identification. By leveraging the self-attention capabilities of vision transformers, we seek to improve the accuracy and robustness of writer identification in historical documents. Vision transformers offer a promising approach for capturing intricate patterns and nuances in handwriting styles, facilitating more nuanced analysis of historical manuscripts. Through rigorous experimentation and validation, our research endeavors to demonstrate the effectiveness of vision transformers in addressing the unique challenges of historical manuscript analysis, contributing to advancements in computational humanities and historical research.

**Figure 1:**
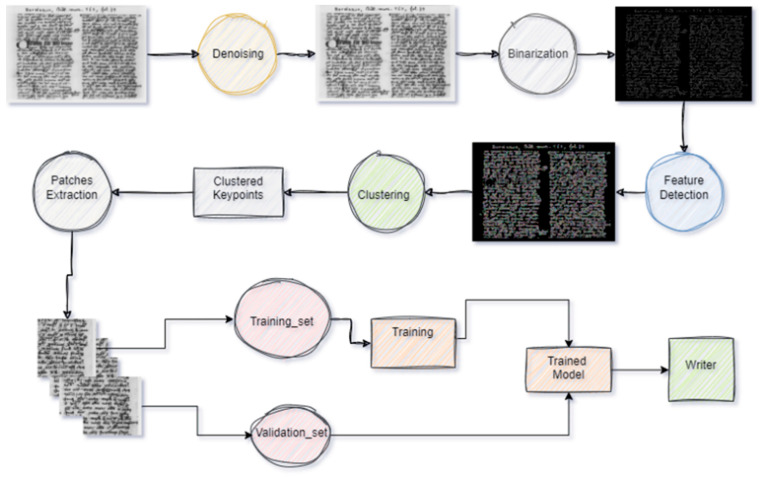
Writer identification system design.

Our approach—as depicted in Figure 1 and inspired by our prior works [29,30]—aims to overcome the shortcomings of previous methods by integrating intelligent feature selection techniques, deep learning architectures, and vision transformers. This integrated methodology comprises three main stages:

- Document preprocessing: Initially, we employ techniques such as denoising and binarization to enhance the quality of manuscript images. This step involves reducing noise and standardizing document representations to establish a robust foundation for subsequent analysis.
- Feature detection: Following preprocessing, we identify and extract meaningful features from the documents. This enables us to characterize distinct handwriting styles and patterns associated with individual writers. Here, we introduce novel feature selection strategies tailored to the nuances of historical manuscript analysis, aiming to capture the intrinsic variability present in handwritten texts.
- Classification: In the final stage, we integrate vision transformers into our writer identification pipeline. Leveraging their self-attention mechanisms, we capture long-range dependencies and contextual information within manuscript images. This allows us to surpass the limitations of traditional convolutional architectures and achieve superior performance in writer identification tasks.

Our contributions extend beyond methodological innovation, aiming to establish a robust framework for historical manuscript analysis and advance the field of computational humanities. Key contributions of this research include the following:

- **Introduction** of a novel approach combining intelligent feature detection techniques and vision transformers, resulting in an efficient system for automated writer identification in historical manuscripts with high accuracy.
- **We employed** effective preprocessing techniques to obtain clean and denoised data, addressing the challenge of limited writing samples by efficiently augmenting our dataset.
- **Additionally**, we utilized automatic keypoints detection combined with k-means clustering to avoid manual region-of-interest selection, ensuring a good distribution of regions throughout the image and minimizing redundancies and irrelevant information.
- **Through** a comprehensive comparative analysis, we evaluated the performance of our proposed system against state-of-the-art methods, justifying our methodological choices and demonstrating the effectiveness of our approach across various experimentation scenarios.

In the subsequent sections of this paper, we delve into a detailed review of related works (Section 2), followed by a comprehensive exposition of our methodology (Section 3) and experimental validation (Section 4). We conclude with a discussion of our findings, and the overarching implications of our research (Section 5).

## 2. Related Works

In this section, we offer an overview of recent research that is pertinent to our study, which centers on the automation of writer identification within historical manuscripts. We delve into various approaches, encompassing both hand-crafted methods and deep learning techniques. Additionally, we explore the transferability of methodologies utilized in contemporary document analysis to historical manuscripts, as well as the potential application of vision transformers in image processing tasks.

Early approaches drew inspiration from contemporary document analysis and subsequently adapted these methodologies for historical documents. Noteworthy studies in this domain, such as those conducted by [1,2,3,4,5], delved into traditional hand-crafted machine learning methods for writer identification. Similarly, several studies applied similar approaches to historical manuscripts, as evidenced by the works of [6,7,8,9,10].

The study outlined in [6] employed image features described using Scale-Invariant Feature Transform (SIFT) features and a visual vocabulary constructed with a Gaussian Mixture Model and Fisher kernel. While originally designed for modern grayscale handwriting, the method encounters challenges with historical manuscripts due to background clutter and water stains. To address this, they adapted the approach for binarized images of ancient writings, resulting in a significant performance. The work presented in [7] introduced multiple methods for extracting features and classifying writers, which proved effective in identifying authors across multipage documents. These methods leverage different principles for feature extraction, such as contour, texture, and keypoint-based approaches, while employing averaging and voting techniques for classification. The experiments conducted using a specialized dataset yielded promising results, with the most successful performance achieved using a novel feature extraction method based on keypoint descriptors. The research presented in [8] introduced novel methods for identifying writers of Arabic historical manuscripts. They proposed techniques that transform keypoint-based features into a global format, capturing broader writing style characteristics. Additionally, they modified common local features, such as contour direction, and demonstrated the effectiveness of combining local and global features for identification. The research also introduced an algorithm used to determine the number of writers involved in manuscript creation. The work found in [9] focused on leveraging handwriting texture as a distinctive attribute of each writer. This was achieved by capturing textural information using oriented Basic Image Features (oBIFs) at different scales. Classification was carried out using various distance metrics, which were then amalgamated to make a final decision through a thorough series of experiments with different oBIF configurations. The work carried out in [10] utilized textural information in handwriting by employing oriented Basic Image Features (oBIFs) and hinge features. Additionally, a novel moment-based matching method was introduced to compare feature vectors extracted from writing samples. Classification was carried out by minimizing a similarity criterion using the proposed moment distance through comprehensive experiments using the ICDAR 2017 dataset.

Despite the automation achieved by these methods, they often yielded suboptimal results, particularly when applied to historical manuscripts. Challenges such as document degradation, varying writing styles, and limited data availability posed significant hurdles to accurate identification. Consequently, there emerged a pressing need for more sophisticated approaches to address these shortcomings. The advent of deep learning methods, particularly convolutional neural networks (CNNs), revolutionized writer identification by leveraging advanced image processing techniques. Studies such as [12,13,14,15] successfully applied CNNs to writer identification tasks, demonstrating improved performance compared to traditional methods. A number of studies applied CNNs to historical [16,17,18,19,20].

In [16], a deep transfer convolutional neural network (CNN) was employed to identify writers using handwriting text line images in English and Arabic. Through transfer learning and data augmentation techniques, they achieve high accuracy rates, with the highest accuracy observed using the freeze Conv5 layer. Their study highlights the effectiveness of deep learning in writer identification tasks. The research published in [17] utilized a three-step model for identifying writers in medieval manuscripts. It employs an object detector to find text lines, a deep neural network for feature extraction and row classification, and a majority vote row-decision combiner for page identification. The approach achieved high accuracy with minimal training data by using MobileNetV2 with SSDLite for text line detection and a meta-architecture classifier for row classification. The study outlined in [18] presented a system tested on a dataset from the Balamand Digital Humanities Center comprising over 11,000 manuscript images, which leverages advanced deep learning techniques. Using SIFT for feature extraction, followed by PCA for dimensionality reduction and k-means clustering for grouping features. A ResNet20 CNN is then trained as a feature extractor. Further feature extraction is done using UBM and k-means clustering. Encoding involves VLAD and PCA. Documents are compared based on the cosine distance between encoded vectors, which is aided by ESVM for final comparison. In [19], three main functions were compared—feature extraction, classification, and encoding, with normalization applied twice. The authors utilized a DeepTen approach integrating feature extraction and encoding with ResNet50 and NetVLAD, respectively. Feature extraction employs a CNN with Spatial Pyramid Pooling, enabling processing of variable-sized inputs. Encoding involves concatenating locally extracted feature vectors and passing them through a NetVLAD network to compute a global descriptor vector. The triplet loss function is employed for training in an end-to-end fashion, optimizing the global descriptors to enforce discriminability between documents. Finally, similarity is computed using cosine distance between global descriptor vectors, with Exemplar SVM used for improved accuracy and precision. In [20], a new deep learning method for identifying writers of historical manuscripts was introduced. It automatically extracts important region of interests from handwritten documents using feature selection method. The process involves cleaning up the images, detecting keypoints, and then classifying them using pretrained CNN models.

Despite the significant progress made in automating writer identification, particularly with the advent of deep learning methods like convolutional neural networks (CNNs), there remain notable challenges, especially in the context of historical manuscripts. These challenges, such as document degradation and variations in writing styles, underscore the need for continued innovation in this field. While CNNs have shown promise in improving identification accuracy, further research is needed to address the complexities inherent in historical documents. In parallel, the field of image processing witnessed the rise of vision transformers as a promising solution for various tasks. Studies like [22,23,24,25,26,31] extensively explored the implementation of vision transformers in different image processing applications, showcasing their effectiveness in capturing complex visual patterns. Despite this progress, no study had investigated the applicability of vision transformers to historical manuscripts. Motivated by this gap in the literature, our study aims to bridge the divide by investigating the potential of vision transformers in automating writer identification in historical manuscripts. By building upon the insights gained from previous research, we propose a novel approach that combines the strengths of deep learning methods with the unique capabilities of vision transformers. Our study represents a culmination of efforts to enhance the automation of writer identification in historical manuscripts, offering a comprehensive and advanced solution to address the challenges inherent in this domain. Through this section, we provide a clear rationale for our research and highlight the contributions of previous studies in shaping our proposed solution.

## 3. Materials and Methods

### 3.1. Dataset

**Figure 2:**
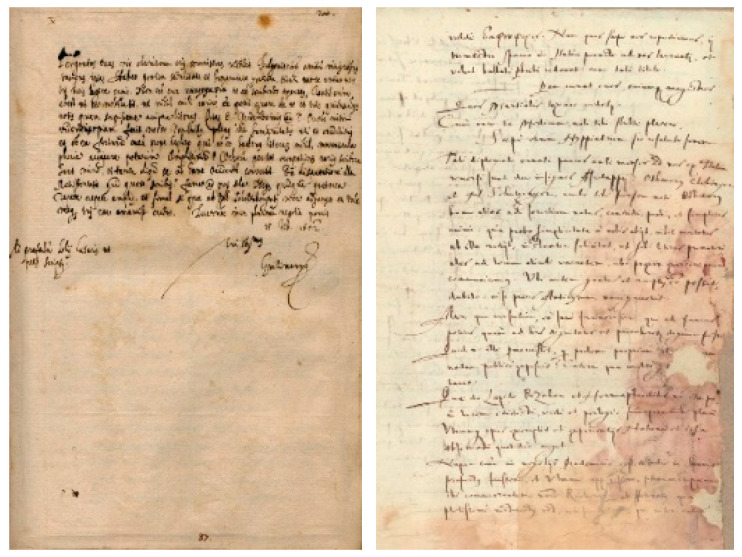
Samples from ICDAR2017-WI dataset.

The dataset utilized in this study originates from the ICDAR2017 competition [32], comprising 3600 samples authored by 720 individuals. Figure 2 shows samples from the dataset. Each writer contributed five documents to the dataset. While this dataset provides a diverse range of handwriting styles, there are notable challenges that necessitate preprocessing. Firstly, the limited number of samples per class (five samples per writer) poses a challenge for training robust identification models. Additionally, a significant portion of the documents within the dataset exhibit large sizes, as depicted in the provided figure. Moreover, many samples suffer from degradation and poor quality, further complicating the training process. Consequently, preprocessing steps are essential to enhance the dataset’s efficiency and suitability for training our writer identification system.

For a relevant categorization, the class labels associated with the charters provided valuable information for training and evaluating machine learning models in the context of manuscript dating by leveraging the MPS dataset in our experiments. Moreover, this open access to the MPS dataset enhanced reproducibility, robust benchmarking, and the ability to make meaningful comparisons between various methodologies. Consequently, we could harness the extensive body of research that has already been undertaken using this dataset, enabling us to evaluate and contrast our findings with those of previous investigations.

### 3.2. Preprocessing

#### 3.2.1. Image Denoising

**Figure 3:**
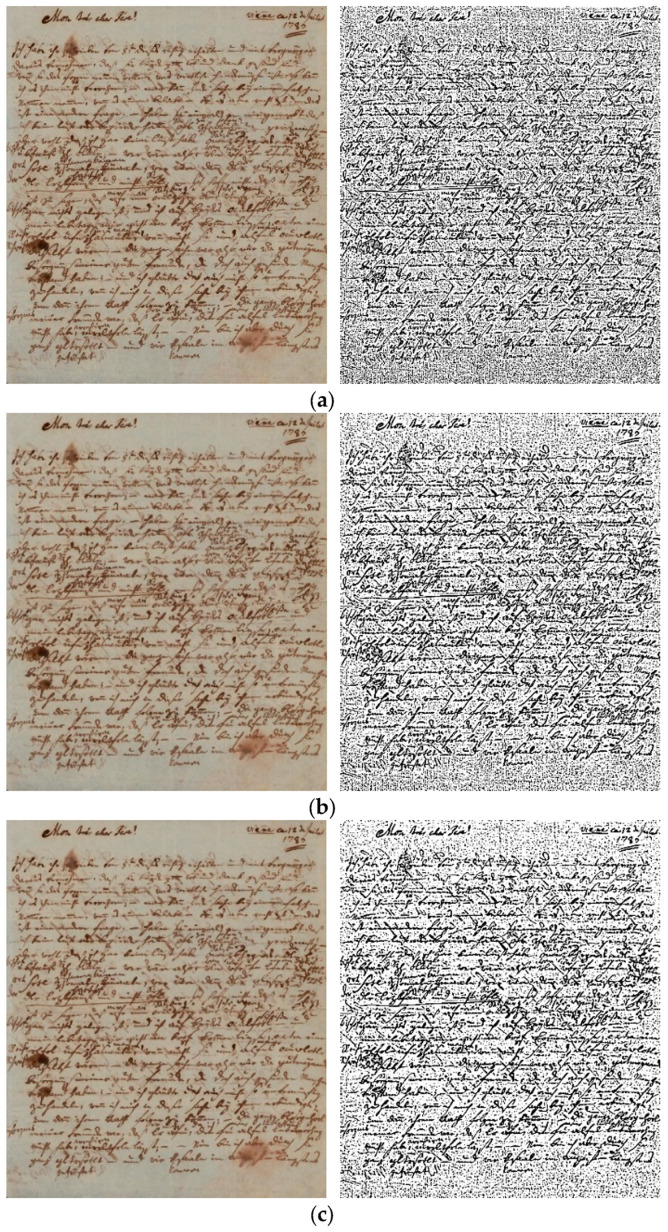

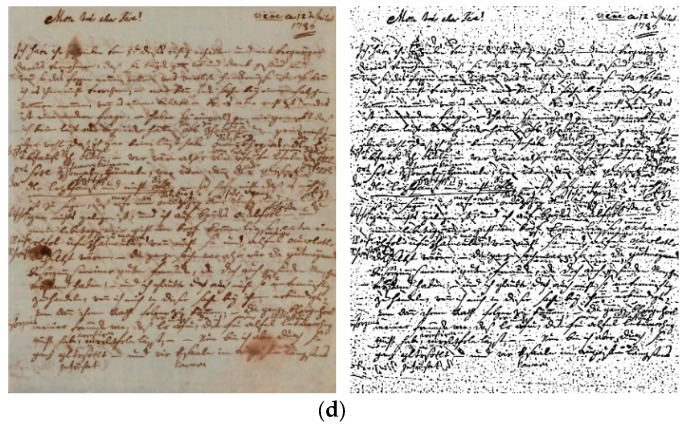
Results of different denoising techniques on a manuscript from the dataset: (**a**) original image with binarization, (**b**) Gaussian filter with binarization, (**c**) median filter with binarization, (**d**) bilateral filtering with binarization.

Given that a significant portion of our dataset exhibits poor quality due to factors such as tear and wear, ink traces, and general degradation, it is imperative to apply denoising techniques to enhance the clarity and legibility of the manuscripts. To address this, we experimented with several existing denoising methods on the original image (Figure 3a), including Gaussian-blur filter (Figure 3b), Median-blur filter (Figure 3c), and bilateral filtering [33] (Figure 3d). After applying each denoising technique to the dataset, we conducted binarization using adaptive thresholding method on the denoised images. This step allowed us to know which method performed well during the denoising process. The results, illustrated in Figure 3, reveal that bilateral filtering outperformed the other denoising techniques considered. This method demonstrated superior performance in preserving important details while effectively reducing noise and enhancing the overall quality of the manuscripts. As a result, bilateral filtering was selected as the preferred denoising method for further preprocessing stages in our writer identification system.

Bilateral filtering plays a crucial role in the classification of historical scripts by effectively addressing various challenges inherent in such documents, which are outlined here as followed:

- Preserving Edges: Bilateral filtering excels in preserving edges within historical scripts, which is essential for maintaining the integrity and legibility of the text during the denoising process. By retaining edge information, the filter ensures that important structural features of the script are not lost, thus aiding in accurate classification.
- Artifact Reduction: Ink traces, worn papers, and other artifacts commonly found in historical manuscripts are effectively reduced through bilateral filtering. By mitigating noise and unwanted elements, such as stains or creases, the filter enhances the clarity of the script, leading to improved classification accuracy.
- Enhancing Feature Extraction: The denoising capabilities of bilateral filtering result in clearer script images, facilitating more efficient feature extraction during subsequent classification stages. By reducing noise and enhancing contrast, the filter enables algorithms to extract meaningful features from the script, which are crucial for accurate writer identification.

Bilateral filtering operates by simultaneously considering spatial and intensity information to determine the weighting of neighboring pixels. The bilateral filter function, denoted as *B*(*x*), is defined by Equation (1):

(1) $$B(x)=\frac{1}{Z}e^{-\frac{{\left\|x-x_{i}\right\|}^{2}}{2\sigma_{s}^{2}}}.e^{-\frac{{\left\|I\left(x\right)-{I(x}_{i)}\right\|}^{2}}{2\sigma_{r}^{2}}}$$

Here, *x* represents the spatial coordinate, *x_i_*, denotes the neighboring pixel, *I*(*x*) is the intensity at position *x*, *σ_s_* is the spatial standard deviation, *σ_r_* is the intensity standard deviation, and *Z* is the normalization constant to ensure proper weighting. The filter operates by considering both the spatial distance between pixels and the difference in intensity values, allowing it to preserve edges while reducing noise effectively.

The filter uses a neighborhood diameter of 9 pixels, a color space sigma (*σ_r_*) of 75, and a coordinate space sigma (*σ_s_*) of 75.

#### 3.2.2. Binarization

In the context of writer identification, binarization serves as a critical preprocessing step aimed at isolating the handwritten text from the background noise in historical manuscripts. By converting grayscale documents into binary images, binarization enhances the clarity and distinction of text regions, which is essential for accurately detecting and analyzing writing styles. Efficient binarization is particularly crucial for writer identification systems, as it enables the identification of unique characteristics and features specific to each writer’s handwriting. By eliminating extraneous elements such as ink traces and background artifacts, binarization helps focus the analysis on the intrinsic properties of the writing itself. It is important to note that binarization was used only to facilitate key point detection, and the binary images were not used directly for training the deep learning model. Therefore, minor binarization imperfections or failure cases had limited impact on the overall classification results.

**Figure 4:**
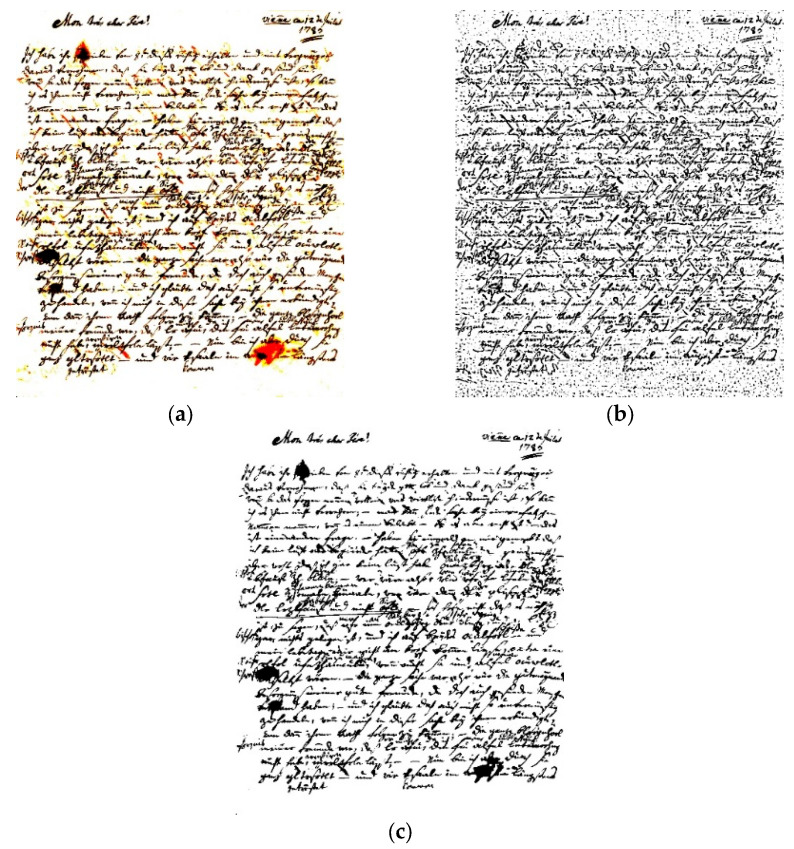
Output of different binarization methods: (**a**) simple threshold, (**b**) adaptive threshold, (**c**) Otsu threshold.

The selection of an appropriate binarization method directly impacts the effectiveness of subsequent feature extraction and classification processes in writer identification. In our study, we evaluated various binarization techniques, including simple threshold (Figure 4a), adaptive threshold (Figure 4b), and Otsu threshold (Figure 4c) [34]. As Figure 4 reveals, we found that the Otsu method consistently produced the most reliable results for our dataset of historical manuscripts. By applying the Otsu method, we ensured that the resulting binary images accurately represented the handwriting patterns of each writer, facilitating more precise feature detection and classification. This enhanced the overall accuracy and reliability of our writer identification system, enabling us to discern subtle differences in writing styles and attribute them to individual authors with greater confidence.

Otsu’s Thresholding Method is based on the principle of maximizing between-class variance to determine an optimal threshold for binarization. Let us denote an input grayscale image I with intensity values ranging from 0 to L − 1, where L is the number of intensity levels. Otsu’s method aims to find a threshold T such that the resulting binary image separates the foreground (script elements) from the background effectively.

The objective function in Otsu’s method in Equation (2) is defined as the weighted sum of variances of the two classes (foreground and background) defined by the threshold T:

(2) $$\sigma_{B}^{2}\left(T\right)=W_{B}\left(T\right).\sigma_{B}^{2}\left(T\right)+W_{F}\left(T\right).\sigma_{F}^{2}(T)$$

where

▪ $\sigma_{B}^{2}\left(T\right)$ and $\sigma_{F}^{2}(T)$ are the variances of the background and foreground classes, respectively.
▪ $W_{B}\left(T\right)$ and $W_{F}\left(T\right)$ are the probabilities of occurrence of the background and foreground classes, respectively.

The objective is to find the threshold *T* that maximizes $\;\sigma_{B}^{2}\left(T\right)$. This can be computed efficiently by iterating through all possible threshold values and calculating the within-class variances and probabilities.

Once the optimal threshold *T* is determined, the image is binarized by assigning pixel values below *T* to the background (0) and pixel values equal to or above *T* to the foreground (1).

Mathematically, the optimal threshold $T_{Otsu}$ can be expressed in Equation (3):

(3) $$T_{Otsu}={\arg max}_{T}\left\{\sigma_{B}^{2}\left(T\right)\right\}$$

*Otsu*’s Thresholding Method effectively enhances the feature detection by accentuating intensity differences between script elements and the background, resulting in clearer and more defined text regions. Its adaptability to varying lighting conditions and intensity distributions makes it particularly suitable for historical document analysis, where such variations are common challenges.

### 3.3. Features Detection

#### 3.3.1. Features from Accelerated Segment Test (FAST)

**Figure 5:**
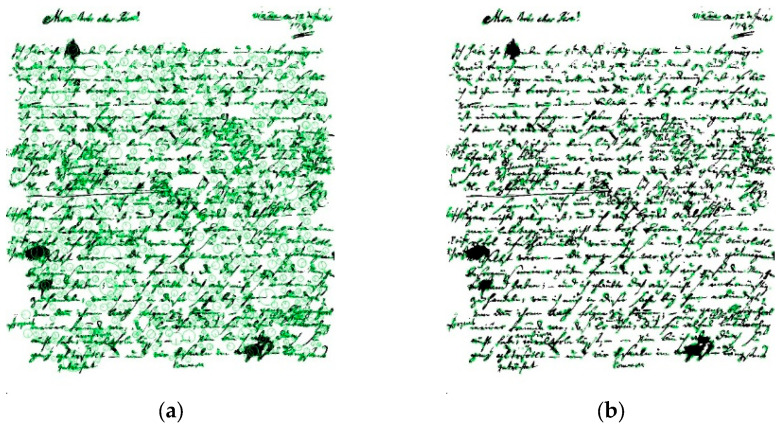
Keypoints detected by (**a**) SIFT detector and (**b**) FAST detector.

In the features detection phase, our goal is to pinpoint key areas within the manuscripts where distinctive writing styles are present. These keypoints serve as reference points for extracting windows that encapsulate unique writing characteristics. By focusing on regions rich in stylistic information, our deep learning model can effectively learn and discern patterns associated with different writers. To achieve this, we explored several feature detection methods, including Scale-Invariant Feature Transform (SIFT) [35], Oriented FAST and Rotated BRIEF (ORB) [36], and Features from Accelerated Segment Test (FAST) [37]. Each of these methods has its strengths and weaknesses, particularly in terms of the number of keypoints detected. In our evaluation, as illustrated in Table 1, we examined the number of keypoints detected by each method. The FAST detector stood out as the fastest among the detectors, identifying a significant number of keypoints (3867) compared to others. Conversely, the SIFT detector yielded a larger number of keypoints (15,156), but Figure 5 highlights that many of these keypoints are redundant. Based on this analysis, we concluded that the FAST method was the most suitable feature detector for our writer identification system.

FAST (Features from Accelerated Segment Test) is a popular algorithm used for corner detection in images. It aims to efficiently identify keypoints, or interest points, in an image by analyzing local intensity variations. In the context of writer identification of historical documents, FAST plays a crucial role in pinpointing regions of the manuscript where distinctive writing styles are present. Mathematically, the FAST algorithm operates by comparing the intensity of a central pixel p to the intensities of pixels on a circle of radius r around it. The algorithm defines a threshold t and checks if the intensity of p is either significantly greater or significantly lower than the intensities of n contiguous pixels on the circle. If n or more consecutive pixels have intensities all greater than p + t or all less than p − t, then p is marked as a corner candidate. Formally, let I(x,y) denote the intensity at pixel (x,y). The algorithm compares the intensity of the central pixel p to the intensities of pixels on the circle of radius r (denoted as p1, p2 …, p6) in a clockwise or counter-clockwise manner. If p is brighter (or darker) than p1 by a threshold t, and at least three consecutive pixels in the circle are brighter (or darker) than p + t (or p − t), then p is classified as a corner.

The key advantage of FAST lies in its computational efficiency, making it suitable for real-time applications. By efficiently identifying keypoints where writing styles are prevalent, FAST enhances the accuracy and efficiency of writer identification systems applied to historical manuscripts.

#### 3.3.2. Clustering and Patch Extraction

Clustering and patch extraction are pivotal steps aimed at enhancing the quality and quantity of data available for model training. Leveraging the keypoints identified by the FAST algorithm, we employed clustering techniques to group these keypoints based on regions of interest within the manuscript images. This process served to eliminate redundancy and ensure diversity in the patches extracted for training our deep learning model.

**Figure 6:**
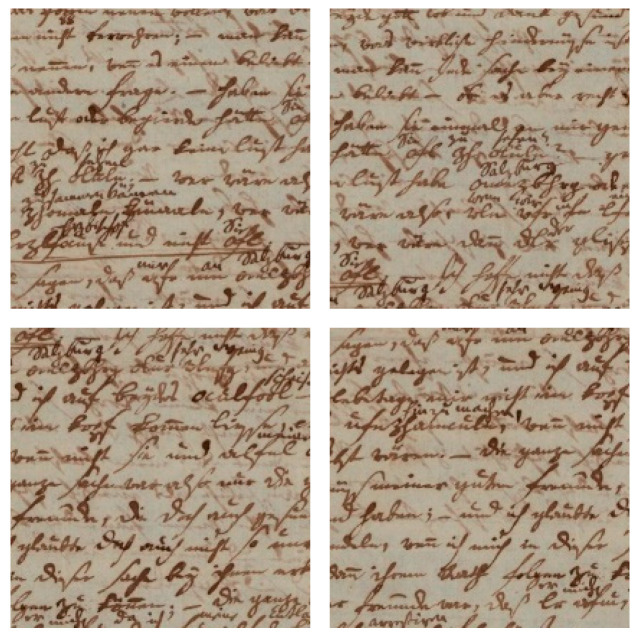
Patches extraction output.

Clustering, particularly using algorithms like k-means [38], allows us to partition the keypoints into distinct clusters, each representing a different region of interest. By selecting windows around each cluster centroid, we can effectively extract patches that encapsulate unique writing styles present in various sections of the manuscript (Figure 6). This approach not only aids in augmenting our dataset but also ensures that the extracted patches offer valuable insights into the diverse writing styles across different regions of the document.

The k-means algorithm iteratively partitions the keypoints into k clusters by minimizing the sum of squared distances between each point and its corresponding cluster centroid. The optimization objective of k-means can be expressed in Equation (4):

(4) $$J=\sum_{j=1}^{k}\sum_{i=1}^{n}{\left\|x_{i}^{\left(j\right)}-c_{j}\right\|}^{2}$$

where

- J represents the objective function, and it quantifies the overall quality of the clustering.
- *K* is the number of clusters that you want to divide your data into.
- *n* is the total number of data points.
- $x_{i}^{\left(j\right)}$ represents the *i*-th data point in the *j*-th cluster.
- $c_{j}$ is the centroid (mean) of the *j*-th cluster.
- ${\left\|x_{i}^{\left(j\right)}-c_{j}\right\|}^{2}$ calculates the squared Euclidean distance between data point $x_{i}^{\left(j\right)}$ and the centroid $c_{j}$ of cluster *j*.

The choice of the number of clusters (*K*) is crucial and is determined dynamically based on the size of each image in our dataset. Instead of fixing a predefined number of clusters, which may lead to redundancies, we devised Equation (5) to determine *K* based on the dimensions of each image. This adaptive approach ensured that the clustering process effectively captured the nuances of different manuscript images, resulting in diverse and informative patches for training.

(5) $$K=\left[\frac{image\_height}{patch\_height}\right]\times \left[\frac{image\_width}{patch\_width}\right]$$

- ***image_height*** and ***image_width*** refer to the dimensions of the original manuscript image.
- ***patch_height*** and ***patch_width*** represent the fixed size of each patch used as input to the vision transformer model.
- The image is divided uniformly into these smaller patches, with no overlap.
- The product of the two ratios gives the total number of patches *K*, which are then treated as input tokens in the transformer architecture.

The choice of the patch size can indeed be determined through experimentation. The appropriate patch size can significantly impact the performance of the clustering and patch extraction process. Therefore, it is essential to conduct experiments to evaluate different patch sizes and select the one that yields the best results for the task at hand. Once the optimal patch size is determined through experimentation, it can be used in conjunction with the equation provided earlier to calculate the number of clusters (*K*). This approach ensures that the clustering process is tailored to the specific characteristics of the dataset and maximizes the effectiveness of feature extraction for subsequent analysis.

This method, summarized in **Algorithm 1**, ensures that patches are evenly distributed across the image while concentrating on visually salient regions.
**Algorithm** **1.** Keypoint-Guided Patch Extraction from Historical Manuscript Images
***Input:***    
*image (grayscale manuscript image)*    
*patch_height, patch_width (desired patch size)*    
*keypoint_detector (e.g., FAST)*
***Output:***
    
*patches[] (list of extracted image patches)*
***1. Preprocess the image:***
    
*a. Apply bilateral filtering for denoising*    
*b. Apply Otsu’s thresholding (optional for clarity)*
***2. Detect keypoints:***
    
*keypoints = keypoint_detector(image)*
***3. Compute the number of clusters:***
    
*K = (image_height // patch_height) × (image_width // patch_width)*
***4. Convert keypoints to coordinates:***
    
*points = [kp.pt for kp in keypoints]*
***5. Apply K-means clustering:***
    
*clusters = KMeans(n_clusters=K).fit(points)*
***6. For each cluster center:***
    
*a. Center a patch of size (patch_height × patch_width) around the cluster center*    
*b. Ensure the patch remains within image bounds*    
*c. Extract the patch and append to patches[]*
***7. Return patches[]***


In summary, our preprocessing pipeline not only enhances the quality and quantity of our dataset but also fosters efficient model training by generating new samples that encapsulate significant information about the various writing styles present in historical manuscripts. This augmentation of the dataset plays a vital role in enabling our deep learning model to learn effectively and accurately identify writers based on their distinct handwriting styles. The selection process for the model and training technique is detailed in the subsequent section.

### 3.4. Training

**Figure 7:**
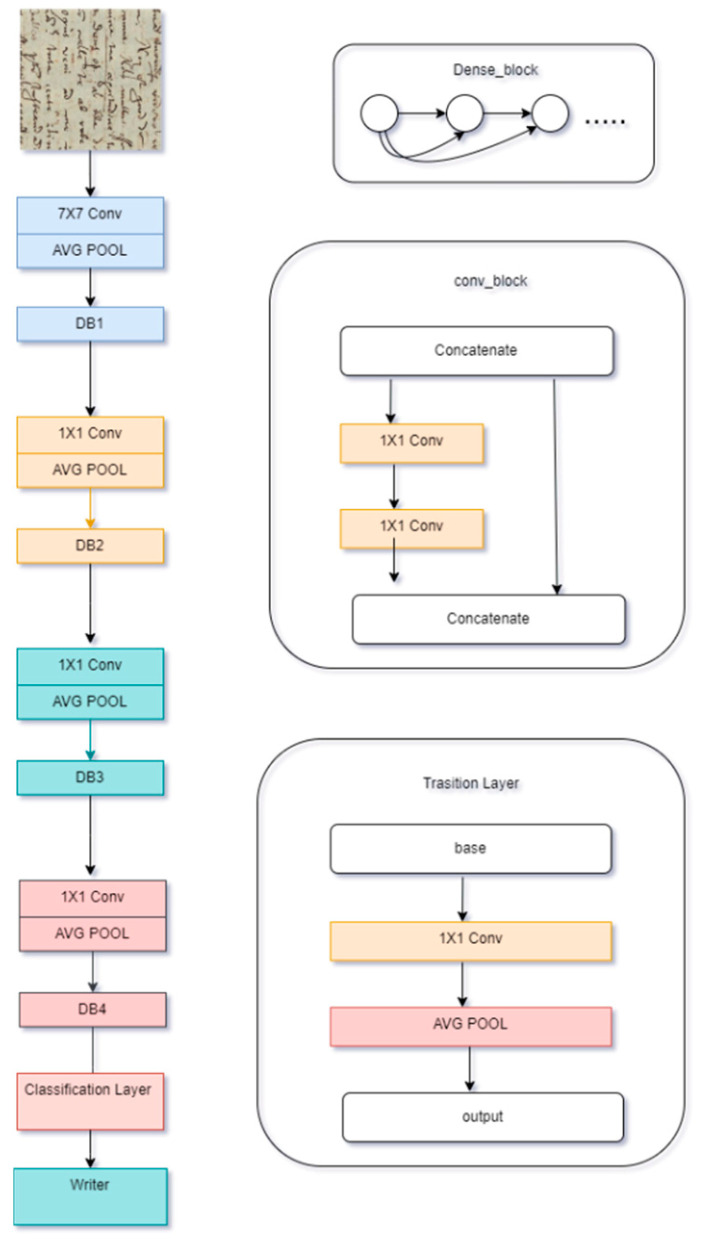
Densenet model architecture [20].

In the realm of historical manuscripts writer identification, the utilization of deep learning techniques has emerged as a cornerstone due to their capacity to learn intricate features, albeit they feature inherent opacity regarding feature learning. This opacity notwithstanding, deep learning stands as a potent tool for capturing complex patterns, particularly crucial for discerning nuances in writing styles. As highlighted in the related work section, numerous studies have leveraged deep learning models, notably convolutional neural networks (CNNs), exemplified by our previous works [20], where we have used Densenet architecture to process our patches (Figure 7). However, with the advent of novel model architectures such as vision transformers, inspired by transformer models in language processing, an opportunity arises to explore their efficacy in historical manuscripts writer identification.

In this context, our writer identification system seeks to assess the effectiveness of vision transformers and their variants. Unlike traditional CNNs, vision transformers offer a unique approach to feature extraction and representation learning, potentially yielding superior performance in capturing intricate writing patterns. Thus, we embark on elucidating the process of training vision transformer models and delineating their architecture, paving the way for a comprehensive understanding of their applicability in historical manuscripts analysis. We will not only investigate the standard ViT architecture [21] but also its variants, namely, Swin Transformer (Swin) [27] and Data-efficient Image Transformer (DeiT) [28]. These variants offer unique features and adaptations that could potentially enhance the performance of our writer identification system.

#### 3.4.1. Vision Transformer (Vit)

**Figure 8:**
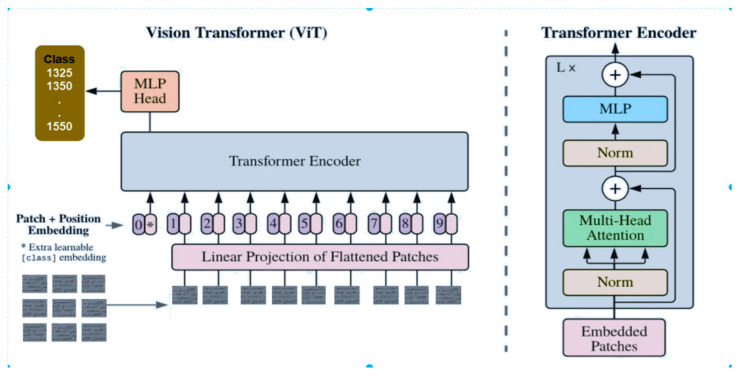
Vision transformer architecture (ViT).

Vision transformers (ViTs) [21] represent a paradigm shift in image processing, offering a unique approach to feature extraction and representation learning. In the context of our historical manuscripts writer identification system, ViTs serve as a compelling alternative to traditional convolutional neural networks (CNNs), offering several advantages in capturing complex patterns inherent in historical scripts. In our system, we aim to harness the power of ViTs to learn discriminative features directly from the sequences of tokens representing manuscript patches. By treating patches as sequences, ViTs enable direct interaction between all regions of the manuscript, allowing the model to capture global context and dependencies crucial for accurate writer identification, as Figure 8 shows. We outline the properties of ViTs in the following:

▪ ViTs utilize self-attention mechanisms to compute attention scores between tokens, determining their importance in relation to each other. This attention mechanism facilitates the aggregation of information from all regions of the manuscript simultaneously, enabling effective feature learning across the entire image.

(6) $$Attention\left(Q,k,V\right)=softmax\left(\frac{{QK}^{T}}{\sqrt{d_{k}}}\right)V$$

- *Q* (Query), *K* (Key), and *V* (Value) are matrices derived from the input patches (tokens) through learned linear projections.
- $QK^{T}$ computes the similarity between each query and all keys, indicating how much focus each token should place on others.
- $\sqrt{d_{k}}$ is a scaling factor (with dkd_kdk being the dimension of the key vectors) that prevents extremely large dot-product values which could push the *softmax* function into regions with very small gradients.
- *softmax* normalizes the similarity scores into attention weights.
- The result is multiplied by *V* (Value) to generate a weighted sum of values, producing context-aware representations for each input token.

Here, *Q*, *K*, and *V* represent the query, key, and value matrices, respectively, which are derived from the input embeddings. $d_{k}$ denotes the dimensionality of the key vectors, and the *softmax* function normalizes the attention scores to obtain a probability distribution over the tokens. We outline additional properties of ViTs in the following:

▪ Moreover, ViTs incorporate multiple layers of self-attention modules, each followed by feedforward neural networks (FFNs), to process and refine the learned features. These FFNs introduce non-linear transformations, enhancing the model’s capacity to capture intricate patterns and nuances present in historical manuscripts.
▪ In our system, we integrate positional encodings into the token embeddings to inject spatial information into the model. This ensures that the ViT architecture can differentiate between tokens representing different spatial locations within the manuscript, further enhancing its ability to understand the structural layout of the text.

By leveraging ViTs in our writer identification system, we aim to exploit their capability to capture global context, learn complex features, and effectively process spatial information, ultimately improving the accuracy and robustness of our model in identifying historical manuscript authors.

#### 3.4.2. Shifted Windows Transformer (Swin)

Swin Transformer [27], short for “Shifted Windows Transformer”, is an attention-based model that excels in capturing long-range dependencies in visual data. Unlike conventional convolutional neural networks (CNNs), which process images using fixed-size convolutional kernels, Swin Transformer adopts a hierarchical structure that dynamically partitions the input image into non-overlapping patches. This approach allows the model to efficiently handle images of arbitrary sizes while maintaining a consistent computational complexity.

**Figure 9:**
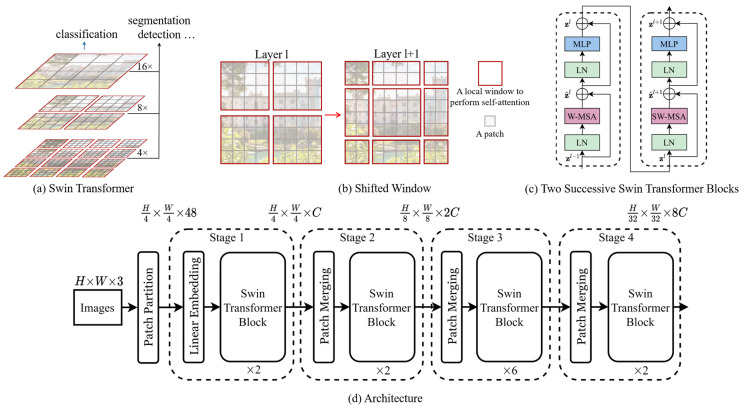
Shifted Windows Transformer architecture (Swin).

The primary distinction between Swin Transformer (Swin) and vision transformer (ViT) lies in their architectural designs and the manner in which they process input data, as shown in Figure 9. Swin Transformer adopts a hierarchical processing approach, dividing the input image into non-overlapping patches at multiple scales. These patches are then hierarchically processed through several stages of transformer blocks, enabling the model to capture both local and global contextual information effectively. In contrast, vision transformer treats the input image as a sequence of flattened patches, processing each patch sequentially through transformer layers. While ViT also captures global dependencies through self-attention mechanisms, it may encounter challenges in handling local context, particularly in high-resolution images. Swin Transformer introduces a window-based self-attention mechanism, grouping tokens into fixed-size windows within each transformer stage. This strategy reduces computational complexity while still enabling the model to capture long-range dependencies efficiently. On the other hand, vision transformer applies standard self-attention mechanisms across all tokens without windowing, which may become computationally expensive for large inputs. Both Swin Transformer and vision transformer utilize positional embeddings to represent the spatial position of each token in the sequence. However, Swin’s hierarchical design may be better suited for capturing complex patterns in large-scale images such as historical manuscripts, owing to its ability to handle both local and global information more efficiently.

In the context of our writer identification system, Swin Transformer offers several advantages over traditional CNNs. Firstly, Swin Transformer’s hierarchical architecture enables it to capture both local and global contextual information from the manuscript images. This is particularly beneficial for writer identification tasks, where subtle writing style cues may span across the entire document. By leveraging self-attention mechanisms, Swin Transformer can effectively integrate information from distant patches, enhancing its ability to discern unique writing patterns.

#### 3.4.3. Data Efficient Image Transformer (DeiT)

**Figure 10:**
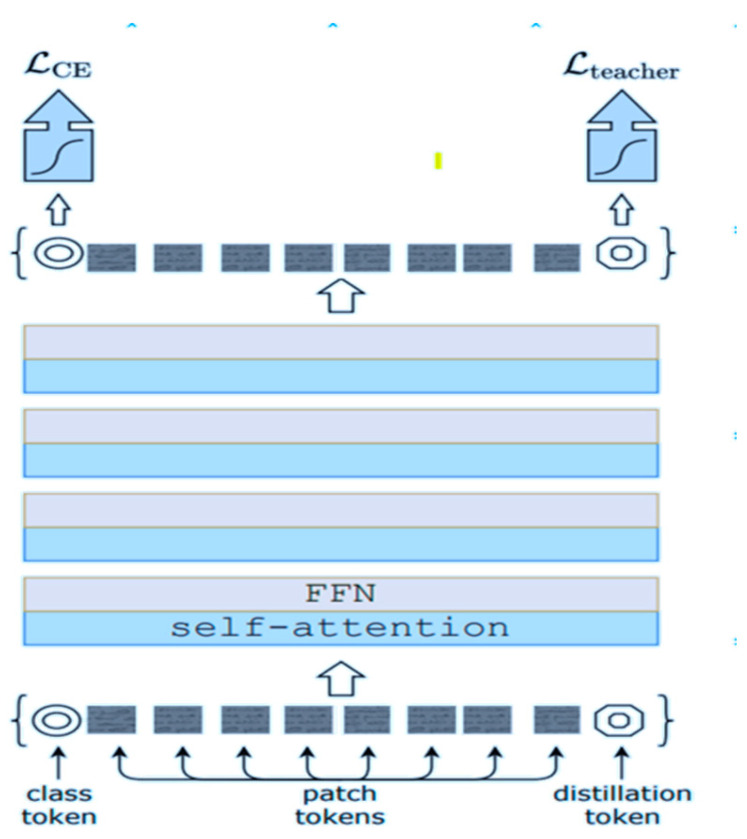
Data-efficient Image Transformer architecture (DeiT).

At the heart of DeiT is the concept of knowledge distillation [28], where a large teacher model pretrained on a vast dataset transfers its knowledge to a smaller student model during training. This distillation process allows the student model to inherit the rich representations learned by the teacher, effectively compressing the knowledge into a more compact form. In our writer identification system, leveraging distillation enables us to train deep transformer models even with limited historical manuscript data, benefiting from the wealth of information distilled from larger pretrained models (Figure 10). Furthermore, DeiT incorporates data augmentation techniques tailored to image classification tasks, such as random cropping, flipping, and color distortion. These augmentations help enrich the training dataset, introducing variations in the input images and enhancing the model’s robustness to different writing styles and document conditions. By augmenting the dataset with diverse transformations, DeiT learns to generalize better from limited training samples, improving its ability to accurately identify writers across a range of historical manuscripts.

When comparing the Data-efficient Image Transformer (DeiT) to the vision transformer (ViT) within the context of writer identification in historical manuscripts, several notable distinctions emerge. DeiT stands out for its enhanced training efficiency, which is achieved through knowledge distillation. Unlike ViT, which typically relies on large-scale datasets for pretraining, DeiT leverages knowledge transfer from a pretrained teacher model to a smaller student model, making it more adept at learning from limited data. Additionally, DeiT exhibits a more compact model size compared to ViT, facilitating faster training times and reduced memory requirements. Through its specialized data augmentation techniques during training, DeiT demonstrates improved generalization and robustness, which are essential for handling variations in writing styles and document conditions inherent in historical manuscripts. Furthermore, DeiT offers greater flexibility in fine-tuning, as its knowledge distillation approach enables effective adaptation to specific writer identification tasks with relatively small datasets.

In terms of computational efficiency, Swin’s sliding window approach allows it to handle larger images more effectively compared to DeiT, which may face challenges with memory constraints when processing high-resolution images due to its attention mechanism operating on the entire image at once. Furthermore, Swin’s hierarchical processing enables it to capture both local and global features effectively, making it well suited for tasks where spatial relationships are crucial, such as identifying intricate details in handwritten historical manuscripts. However, DeiT’s knowledge distillation approach and data-efficient training make it more adaptable to scenarios where computational resources or training data are limited.

The selection between Vit, DeiT, and Swin models for our writer identification system in historical manuscripts was ultimately determined through rigorous experimentation, as will be detailed in the upcoming experiment section. By empirically evaluating the performance of these models on our dataset and considering factors such as computational efficiency, accuracy, and adaptability to data constraints, we were able to make an informed decision regarding the most suitable architecture. This experimental approach ensured that the chosen model aligned closely with the specific requirements and challenges posed by historical manuscript analysis, leading to an optimized and effective writer identification system.

## 4. Experiments and Results

Our writer identification system underwent a meticulous optimization process involving numerous parameters and hyperparameters across various development phases. Beginning with preprocessing section, we explored different techniques to enhance the quality of manuscript images. These included experimenting with denoising methods, binarization techniques, and other image enhancement approaches to prepare the data for subsequent analysis. Following preprocessing, we delved into feature detection methods, evaluating the effectiveness of algorithms like FAST, SIFT, and ORB in capturing key writing style features while minimizing noise and redundancy.

In this section, our focus shifts to training, where we fine-tuned our system’s parameters. This involved experimenting with different vision transformer models like (ViTs), Swin, DeiT, and its variants. These experiments covered a range of aspects, including hyperparameters fine-tuning, preprocessing effects, patch size, and model selection. To accelerate experimentation, we focused on one patch from each document, ensuring efficient evaluation while maintaining representative samples. The primary evaluation metrics used throughout these experiments were accuracy metrics (top1, top5, and top10), providing a quantitative measure of our system’s performance.

For each experiment, the dataset was randomly divided into training and validation sets, with 80% of the patches allocated for training and 20% for validation. After fine-tuning our system parameters and selecting the optimal configurations, we obtained the results of our final system. These include accuracy metrics (top1, top5, and top10) and comparisons with similar studies. By systematically exploring different parameters and evaluating their impact on performance, we aim to build a robust and effective writer identification system capable of accurately analyzing historical manuscripts.

### 4.1. Model Hyperparameters

The objective of this experiment was to determine the optimal hyperparameters for training our writer identification system. Hyperparameters such as the learning rate and optimizer significantly influence the model’s performance and generalization ability. This streamlined approach allowed us to efficiently explore various hyperparameter configurations and their impact on the model’s performance.

By systematically exploring different hyperparameter values and evaluating their impact on model performance, we could identify the optimal configuration for training our writer identification system. This iterative process allowed us to refine the hyperparameter values based on observed results, ultimately improving the model’s accuracy and generalization ability. Through this experiment, we gained insights into the influence of hyperparameters on the classification framework, enabling us to make informed decisions for future model training. Table 2 presents the results of the experiments, showcasing the performance of various hyperparameter combinations.

The results of the experiments reveal the significant impact of hyperparameters on the performance of our writer identification system.

For a learning rate of 0.01 with the Adam optimizer, the system achieved a Top-1 accuracy of 0.92%, Top-5 accuracy of 3.71%, and Top-10 accuracy of 7.43%. This combination demonstrates decent performance across all metrics.

However, when the learning rate was reduced to 0.001 with the Adam optimizer, there was a notable increase in performance, with a Top-1 accuracy of 26.51%, Top-5 accuracy of 51.39%, and Top-10 accuracy of 62.36%. This suggests that a lower learning rate results in significantly improved performance, particularly in terms of Top-1 accuracy.

On the other hand, when using the SGD optimizer with a learning rate of 0.01, the system achieved even higher accuracy, with a Top-1 accuracy of 79.02%, Top-5 accuracy of 88.83%, and Top-10 accuracy of 91.46%. This indicates that the choice of optimizer, in combination with an appropriate learning rate, plays a crucial role in determining system performance.

When the learning rate was reduced to 0.001 with the SGD optimizer, there was a significant decrease in performance compared to the higher learning rate, with a Top-1 accuracy of 61.83%, Top-5 accuracy of 79.81%, and Top-10 accuracy of 86.34%. However, these results still outperformed the initial combination of 0.001 learning rate with the Adam optimizer.

Overall, these findings underscore the importance of carefully selecting hyperparameters to optimize the performance of the writer identification system. Lower learning rates generally led to improved accuracy, with the choice of optimizer also exerting a significant influence on system performance. Further experimentation and fine-tuning of hyperparameters may yield even better results and enhance the overall effectiveness of the system.

### 4.2. Impact of Preprocessing

Table 3 provides a detailed overview of the experiments conducted to assess the impact of preprocessing techniques on the performance of our writer identification system. The experiments were organized into distinct phases, each targeting specific preprocessing strategies to elucidate their influence on model performance. In the following, we elucidate the experiments in order of the phases through which they were conducted:

- **Baseline Phase:** In the initial phase, no preprocessing techniques were applied, and the images were solely resized to match the model’s input dimensions. This served as a baseline to gauge the model’s performance without any additional preprocessing.
- **Random Patch Extraction:** The subsequent phase involved extracting random patches of uniform dimensions from the images. Unlike the baseline approach, which resized entire images, this strategy randomly sampled patches, introducing variability and diversity into the experiment.
- **Preprocessing:** In the final phase, patches were extracted based on regions of interest detection. Keypoints were identified using the FAST detector, followed by centroid extraction through k-means clustering. This meticulous process ensured that patches were selected from regions specifically adorned with text, enhancing the relevance of extracted features for writer identification.

The rationale behind these experiments lies in the deliberate exploration of preprocessing techniques to enhance the discriminative power of the model. Each phase contributed valuable insights, with the final phase focusing on extracting patches from text-rich regions, thereby amplifying the relevance of features for writer identification. Through this strategic continuum of experimentation, we aimed to unravel the nuanced impact of preprocessing on the efficacy of our writer identification system.

The results of the experiments comparing identification performance with and without preprocessing steps highlight the crucial role of preprocessing in enhancing the accuracy of the writer identification system.

**Without Preprocessing (Only Resizing):** The system achieved relatively low accuracy when no preprocessing steps were applied, with a Top-1 accuracy of 17.53%, Top-5 accuracy of 31.07%, and Top-10 accuracy of 35.93%. This indicates that simply resizing the images without any further preprocessing is insufficient for effective writer identification.

**Random Patches:** Introducing random patches led to a significant improvement in identification performance. The Top-1 accuracy jumped to 59.84%, Top-5 accuracy to 77.17%, and Top-10 accuracy to 83.54%. This suggests that extracting patches from the images allows the system to focus on localized regions containing relevant information for writer identification.

**With Preprocessing:** Implementing preprocessing steps such as denoising and binarization and then extracting patches based on detected region of interests yielded the highest accuracy among all approaches. The Top-1 accuracy improved substantially to 79.02%, with the Top-5 accuracy at 88.83% and Top-10 accuracy at 91.46%. This underscores the importance of preprocessing in enhancing the quality of the input data and facilitating more accurate feature extraction and classification.

Overall, these results demonstrate the significant impact of preprocessing on the performance of the writer identification system. Preprocessing techniques such as denoising, binarization, feature detection, and patch extraction helped to enhance the clarity and quality of the manuscript images, enabling the system to extract more meaningful features and achieve higher accuracy in identifying writers. Therefore, preprocessing should be considered an essential step in the pipeline of our writer identification system aiming for optimal performance.

### 4.3. Impact of Patch Size

In this experiment, we aimed to determine the optimal patch size for extracting features from manuscript images within our writer identification system. Our objective was to assess how different patch sizes influenced the performance of our model in accurately identifying writers based on their handwriting styles. We conducted a series of experiments using various patch sizes, including 150 × 150, 224 × 224, 550 × 550, and 750 × 750 pixels, as detailed in Table 4.

These experiments were carried out using the same model architecture and hyperparameters as in previous iterations, ensuring consistency and comparability of the results. By systematically varying the patch sizes and evaluating the model’s performance, we sought to identify the patch size that maximized the extraction of relevant features crucial for writer identification. The results obtained from these experiments will provide valuable insights into the impact of patch size on the effectiveness of our writer identification system. Specifically, we aimed to determine the patch size that optimally captured the nuances of the handwriting styles present in the manuscript images. This knowledge will inform our decision-making process, allowing us to select the most suitable patch size for achieving high accuracy in writer identification.

Furthermore, once the optimal patch size is identified, we will extend our approach to extract patches from all regions of interest within the manuscript images. This comprehensive strategy will enable us to leverage the full potential of our methodology, enhancing the accuracy and robustness of our writer identification system.

The results presented in Table 4 demonstrate the impact of patch size on the identification performance of the system, highlighting the importance of selecting an appropriate patch size for optimal results. The results for each patch size were as follows:

◦ **150 × 150 Patches:** Using smaller patches of size 150 × 150 pixels yielded moderate identification performance, with a Top-1 accuracy of 55.25%, Top-5 accuracy of 78.16%, and Top-10 accuracy of 86.06%. This suggests that while smaller patches may capture finer details, they may also lack sufficient contextual information for accurate writer identification.
◦ **224 × 224 Patches:** Increasing the patch size to 224 × 224 pixels resulted in improved identification performance across all metrics. The Top-1 accuracy increased to 66.79%, with the Top-5 accuracy at 84.98% and Top-10 accuracy at 90.45%. This suggests that larger patches provide a better balance between capturing detailed features and preserving contextual information.
◦ **550 × 550 Patches:** Optimal identification performance was achieved with patches of size 550 × 550 pixels. The system achieved its highest accuracy, with a Top-1 accuracy of 79.02%, Top-5 accuracy of 88.83%, and Top-10 accuracy of 91.46%. This indicates that patches of this size effectively capture both detailed features and contextual information, facilitating more accurate writer identification.
◦ **750 × 750 Patches:** Using larger patches of size 750 × 750 pixels led to a slight decrease in identification performance compared to the optimal patch size. The Top-1 accuracy dropped to 59.54%, with the Top-5 accuracy at 76.98% and Top-10 accuracy at 81.39%. This suggests that larger patches may introduce more irrelevant information or dilute the importance of key features, leading to reduced accuracy.

In summary, the results underscore the importance of selecting an appropriate patch size for effective writer identification. Patches of size 550 × 550 pixels exhibited the best balance between capturing detailed features and preserving contextual information, resulting in optimal identification performance.

### 4.4. Model Selection

In this phase, our focus shifted to comparing the performance of multiple pretrained vision transformers within our writer identification system. Our objective was to evaluate how different pretrained models performed when applied to our specific task and dataset. To achieve this, we trained several models using identical hyperparameters, with SGD serving as the optimizer and with a fixed learning rate of 0.01 for 20 epochs. The results of these experiments are presented in Table 5, showcasing the performance metrics of each pretrained vision transformer model. Through this comparison, we sought to discern which model exhibits the highest accuracy and effectiveness in identifying writers based on their handwriting styles. By carefully analyzing the results, we could determine the most suitable pretrained model for integration into our writer identification system. This informed selection will guide us in choosing the optimal model architecture to further refine and enhance the performance of our writer identification system.

The results presented in Table 5 illustrate the performance of different pretrained models on the writer identification task, shedding light on the effectiveness of each model in capturing relevant features and achieving accurate classification.

**Vit-Ti:** The Vit-Ti model demonstrated solid performance across all metrics, with a Top-1 accuracy of 72.85%, Top-5 accuracy of 86.72%, and Top-10 accuracy of 89.46%. Despite being the smallest variant of the vision transformer architecture, Vit-Ti achieved competitive results, indicating its suitability for the writer identification task.

**Vit-S:** The Vit-S model exhibited improved performance compared to Vit-Ti, with a Top-1 accuracy of 79.02%, Top-5 accuracy of 88.83%, and Top-10 accuracy of 91.46%. The larger size and capacity of Vit-S likely contributed to its enhanced capability to capture intricate patterns and nuances in handwriting styles.

**Vit-B:** Vit-B performed comparably to Vit-Ti, with a Top-1 accuracy of 73.27%, Top-5 accuracy of 86.51%, and Top-10 accuracy of 89.35%. Despite its larger size relative to Vit-Ti, Vit-B achieved similar results, suggesting that its additional parameters may not have significantly enhanced its performance for this task.

**DeiT-S:** The DeiT-S model achieved competitive performance, with a Top-1 accuracy of 75.75%, Top-5 accuracy of 87.46%, and Top-10 accuracy of 89.99%. The results for DeiT-S demonstrate its adaptability and effectiveness in writer identification, showcasing its versatility across different domains.

**Swin-S:** Swin-S emerged as the top-performing model in this comparison, boasting a Top-1 accuracy of 80.50%, Top-5 accuracy of 90.04%, and Top-10 accuracy of 91.83%. The Swin Transformer’s unique architecture, featuring hierarchical partitioning of images, proved highly effective in capturing spatial dependencies and contextual information, leading to superior performance in writer identification.

In summary, the choice of pretrained model significantly impacted the performance of the writer identification system. Swin-S emerged as the most effective model in this evaluation, outperforming other variants of vision transformers and demonstrating the importance of architectural design in achieving accurate classification results.

### 4.5. Final System Results

The final configuration of our writer identification system is presented in Table 6. We employed Stochastic Gradient Descent (SGD) as the optimizer, with a learning rate of 0.01, a batch size of 32, and patches of size 550 × 550 pixels. The chosen model for this configuration was Swin-S, which demonstrated superior performance in our previous experiments. The evaluation metrics for the final system, including Top-1, Top-5, and Top-10 accuracies, are also provided in the table.

We manually reviewed a sample of misclassified instances and observed that the model tended to struggle in the following scenarios:

- Highly similar handwriting styles between different writers, especially in short texts.
- Degraded or low-contrast manuscript images, where noise overwhelms distinguishing features.
- Sparse keypoint distributions in pages with limited handwriting content, leading to insufficient patch diversity.

### 4.6. Performance Comparison

Table 7 presents a comparison of the performance of our proposed writer identification system with other existing methods:

Our proposed method outperforms most of the existing techniques in terms of both Top-1 and Top-10 accuracy. It features a significantly higher accuracy of 90.59 in Top-1 identification and 94.57 in Top-10 identification, showcasing the effectiveness and robustness of our approach. Comparatively, other methods, such as AngU-Net + R-SIFT, SIFT + NBNN, and SRS-LBP, demonstrate lower accuracy scores, indicating their limitations in accurately identifying writers in historical manuscripts. Additionally, our method surpasses traditional techniques like Harris + CNN and moment-based distance features matching by a considerable margin, highlighting the advancements made possible through the integration of intelligent feature selection methods and deep learning architectures in our system. This comparison demonstrates the superior performance of our proposed writer identification system in accurately identifying writers in historical manuscripts.

### 4.7. Limitations and Future Work

While our proposed system demonstrates promising performance in historical writer identification using vision transformers and intelligent feature selection, several limitations should be acknowledged. First, due to computational constraints, we conducted a single run for each experimental configuration and did not report standard deviations or confidence intervals. Although the observed improvements were consistent across model variations, the absence of multiple-run statistics limits our ability to assess the stability and statistical significance of the results. Future work will address this limitation by incorporating repeated trials and cross-validation strategies to more rigorously evaluate the robustness of the system.

Second, the experiments were conducted exclusively on the ICDAR2017-WI dataset, which may limit the generalizability of our findings to broader historical manuscript collections with varying scripts, styles, and periods. Evaluating the proposed system on additional datasets is necessary to confirm its applicability across diverse archival scenarios.

Third, while we justified the use of the FAST detector based on its speed and reduced redundancy compared to alternatives like SIFT or ORB, we did not perform an end-to-end classification accuracy comparison between these detectors. Due to the high computational cost associated with retraining transformer-based models, such experiments were beyond the scope of this study. However, we recognize the value of these comparisons and propose them as a direction for future research, ideally through targeted ablation studies on representative subsets of the data to assess the tradeoffs between computational efficiency and classification accuracy.

## 5. Conclusions

In conclusion, this study presents a novel system for writer identification in historical manuscripts, leveraging intelligent feature selection methods with vision transformers. Our findings underscore the efficacy of our system in accurately identifying writers based on their distinct handwriting styles. The methodology comprises three main steps:

- Preprocessing: Our approach begins with preprocessing the historical manuscripts, involving bilateral filtering for denoising and Otsu thresholding for binarization. These techniques enhance the quality of the documents, preparing them for subsequent feature extraction.
- Feature Detection: We utilize the FAST method to detect keypoints within the manuscripts, followed by clustering using k-means. This process facilitates the extraction of regions of interest in the form of patches with uniform sizes, optimizing feature representation for classification.
- Classification: The extracted patches are then classified using vision transformer architectures, including VIT, Swin, and Deit. These deep learning models effectively classify the patches based on learned features, enabling accurate writer identification.

Through a series of experiments, we meticulously fine-tuned our system parameters, including hyperparameters, patch sizes, and model selection. This iterative process allowed us to optimize our system’s performance and ensure robustness across different configurations. Subsequently, we trained our system using these optimized parameters to obtain the final results. Our study yields several significant results, including the following:

- The effectiveness of our preprocessing techniques in enhancing the quality of historical manuscripts.
- The efficacy of our intelligent feature selection method in extracting informative regions from the documents.
- The superior performance of vision transformer architectures in classifying manuscript patches compared to traditional methods.

Furthermore, our comparative analysis demonstrates the superiority of our approach over state-of-the-art studies in writer identification. However, it is essential to acknowledge the limitations of our study, primarily its performance on a specific dataset rather than generalizing across multiple datasets. Therefore, as future work, we aim to enhance our system’s adaptability to various datasets and develop user-friendly software with intuitive interfaces. Additionally, we plan to extend our system’s capabilities to other tasks in historical manuscript analysis, further advancing the field. In conclusion, our novel approach has shown promising results in the analysis of historical manuscripts, paving the way for future advancements in this domain.

## Figures and Tables

**Table 1:** Comparison of various feature detection methods.

Features-Detector	Time (Seconds)	Keypoints Number
FAST	0.00001	3876
**SIFT**	**0.2449**	**15,156**
ORB	1.78	500

**Table 2:** Performance with various combinations of model hyperparameters.

Learning Rate	Optimizer	Top-1	Top-5	Top-10
0.01	Adam	0.92	3.71	7.43
0.001	Adam	26.51	51.39	62.36
**0.01**	**SGD**	**79.02**	**88.83**	**91.46**
0.001	SGD	61.83	79.81	86.34

**Table 3:** Identification performance with and without preprocessing steps.

Experiment	Top-1	Top-5	Top-10
Without Preprocessing (Only resizing)	17.53	31.07	35.93
Random patches	59.84	77.17	83.54
**With preprocessing**	**79.02**	**88.83**	**91.46**

**Table 4:** Identification performance as a function of patch size.

Patches Size	Top-1	Top-5	Top-10
150 × 150	55.25	78.16	86.06
224 × 224	66.79	84.98	90.45
**550 × 550**	**79.02**	**88.83**	**91.46**
750 × 750	59.54	76.98	81.39

**Table 5:** Performance on different pretrained models.

Model	Top-1	Top-5	Top-10
Vit-Ti	72.85	86.72	89.46
Vit-S	79.02	88.83	91.46
Vit-B	73.27	86.51	89.35
DeiT-S	75.75	87.46	89.99
**Swin-S**	**80.50**	**90.04**	**91.83**

**Table 6:** Final system parameters.

Optimizer	Learning Rate	Batch Size	Patch Size	Model	Top-1	Top-5	Top-10
SGD	0.01	32	550 × 550	Swin-s	90.59	94.08	94.57

**Table 7:** Performance Comparison.

Study		Top-1	Top-10
**Proposed method**		**90.59**	**94.57**
Harris + CNN	[20]	85.2	93.6
VLAD-encoded CNN features	[32]	88.9	93.8
SIFT and pathlet features with bagged-VLAD	[39]	90.1	-
VLAD-encoded CNN features with reranking	[40]	89.43	-
Moment-based distance features matching	[8]	78.75	91.08
Combined basic image features	[9]	77.39	-
OBIF	[32]	76.4	-
SRS-LBP		67.0	80.1
SIFT + NBNN		67.1	80.2
CoHinge features		76.1	85.8
AngU-Net + R-SIFT		48	92

## Data Availability

The data that support the findings of this study are openly available in Zenodo at https://zenodo.org/records/5527690 (accessed on 6 June 2025).
